# JunD/AP-1-Mediated Gene Expression Promotes Lymphocyte Growth Dependent on Interleukin-7 Signal Transduction

**DOI:** 10.1371/journal.pone.0032262

**Published:** 2012-02-23

**Authors:** Shannon M. Ruppert, Mounir Chehtane, Ge Zhang, Haiyan Hu, Xiaoman Li, Annette R. Khaled

**Affiliations:** 1 From the Burnett School of Biomedical Sciences, College of Medicine, University of Central Florida, Orlando, Florida, United States of America; 2 Department of Electrical Engineering and Computer Science, University of Central Florida, Florida, United States of America; Institut national de la santé et de la recherche médicale - Institut Cochin, France

## Abstract

Interleukin-7 (IL-7) is an essential cytokine for lymphocyte growth that has the potential for promoting immune reconstitution. This feature makes IL-7 an ideal candidate for therapeutic development. As with other cytokines, signaling through the IL-7 receptor induces the JAK/STAT pathway. However, the broad scope of IL-7 regulatory targets likely necessitates the use of other signaling components whose identities remain poorly defined. To this end, we used an IL-7 dependent T-cell line to examine how expression of the glycolytic enzyme, Hexokinase II (HXKII) was regulated by IL-7 in a STAT5-independent manner. Our studies revealed that IL-7 promoted the activity of JNK (Jun N-terminal Kinase), and that JNK, in turn, drove the expression of JunD, a component of the Activating Protein 1 (AP-1) transcription factors. Gel shifts showed that the AP-1 complex induced by IL-7 contained JunD but not c-Fos or c-Jun. Inhibition of JNK/JunD blocked glucose uptake and HXKII gene expression, indicating that this pathway was responsible for promoting HXKII expression. Because others had shown that JunD was a negative regulator of cell growth, we performed a bioinformatics analysis to uncover possible JunD-regulated gene targets. Our search revealed that JunD could control the expression of proteins involved in signal transduction, cell survival and metabolism. One of these growth promoters was the oncogene, Pim-1. Pim-1 is an IL-7-induced protein that was inhibited when the activities of JNK or JunD were blocked, showing that in IL-7 dependent T-cells JunD can promote positive signals transduced through Pim-1. This was confirmed when the IL-7-induced proliferation of CD8 T-cells was impaired upon JunD inhibition. These results show that engagement of the IL-7 receptor drives a signal that is more complex than the JAK/STAT pathway, activating JNK and JunD to induce rapid growth stimulation through the expression of metabolic and signaling factors like HXKII and Pim-1.

## Introduction

Interleukin-7 (IL-7) plays a major role in lymphocyte survival, development and proliferation [1]. Because of its importance as a lymphocyte growth factor, IL-7 has potential use as a therapeutic agent in cancer therapy [2], bone marrow transplantation [3] and treatment of infectious diseases like sepsis [4] and HIV [5]. IL-7 is a 25 kDa protein that is not produced by lymphocytes but was discovered as a product of a thymic stromal cell line [6]. Tissues that produce IL-7 include the generative lymphoid organs [7], [8]; however, the regulation of IL-7 production remains unclear but could be induced in nonlymphoid tissues upon infection [9]. The receptor for IL-7 (IL-7R), expressed by lymphocytes, consists of the IL-7Rα chain and the common cytokine γ chain (γc) [10]. Upon binding of IL-7, the two receptor chains heterodimerize and initiate signaling mediated through receptor-associated kinases, Janus kinases, Jak1/Jak3, which phosphorylate and activate the transcription factor, STAT5a/b [1]. The most recent biological information on IL-7 signal transduction focuses on the JAK/STAT pathway, with scant new information on probable crosstalk with other signaling pathways.

Descriptions of the IL-7R interacting with Fyn or Lyn were published in the early 1990's [11], [12], while other early work revealed aspects of Mitogen Activated Protein Kinase (MAPK) signaling in the context of IL-7 [13], [14]. Lck-mediated T-cell receptor (TCR) signaling was shown to both synergize and operate independently of IL-7 signaling to promote T-cell proliferation [15]. We found that inhibition of Lck modulated the IL-7 dose response of CD4 but not CD8 T-cells by regulating the activation of STAT5 [16]. In regards to the MAPKs, IL-7 could induce T cell proliferation in the absence of ERK (Extracellular signal-Regulated Kinase) activity [13] but required p38 MAPK [14]. In contrast, we and others reported that IL-7 withdrawal induced the activity of p38 MAPK and JNK (Jun N-terminal Kinase) and promoted apoptosis [17], [18] as well as cell cycle arrest [19]. How MAPKs, like p38 MAPK and JNK, can have both growth-promoting and apoptotic functions that are controlled by IL-7 remains unknown.

One of the functions of the MAPK family is the post-translational regulation of Activating Protein 1 (AP-1) transcription factors. AP-1 are dimeric transcription factors composed of proteins with basic leucine zipper domains needed for dimer formation and DNA-binding [20]. The major subfamilies that form AP-1 are Jun (c-Jun, JunB and JunD) and Fos (c-Fos, FosB, Fra1 and Fra2). Activating Transcription Factor (ATF) proteins and Maf proteins can also be components [21]. The potential for multiple combinations gives the AP-1 family its specificity and capacity for a large repertoire of regulated-genes [22]. The activity of AP-1 is mediated in part by ERK, JNK, or FRK (Fyn-Related Kinase) - each of these kinases phosphorylates different substrates. Three distinct JNK gene families have been described: JNK1, JNK2, and JNK3. JNK1 and 2 are ubiquitously expressed, while JNK3 is selectively expressed in neuronal and cardiac tissues [23]. The Jun family is activated through phosphorylation by JNKs, with c-Jun and JunD being recognized targets [24]. While c-Jun and JunB are considered activating factors and expressed as early genes [25]–[27], JunD was first described as a negative regulator of cell proliferation [28]–[30].

There exists a number of IL-7 functional targets [31]. To name a few, IL-7 promotes cell survival through Bcl-2 upregulation and repression of Bax [32]–[34], Bim [35] or Bad [36]. IL-7 drives proliferation through cell cycle regulators such as Cdc25A [19] and p27 kip [37]. IL-7 also regulates glucose metabolism [34], [38], and this function is partially fulfilled through transcriptional control of Hexokinase II (HXKII) [39]. How other signaling pathways augment the JAK/STAT signal and contribute to T-cell growth is unknown. In this study, we investigated the activity of the JNK pathway in IL-7- dependent T-cells and found that it involved the activation of JunD-containing AP-1 complexes. JunD/AP-1 drove the expression of HXKII. Using a bioinformatics approach, we discovered other JunD-regulated genes that included the oncogene, Pim-1, which was induced by IL-7. The importance of these findings is the demonstration that, in T-cells, JunD can modulate positive growth in response to IL-7, enhancing the initial cytokine signal transduced by JAK/STAT.

## Results

**Figure 1:**
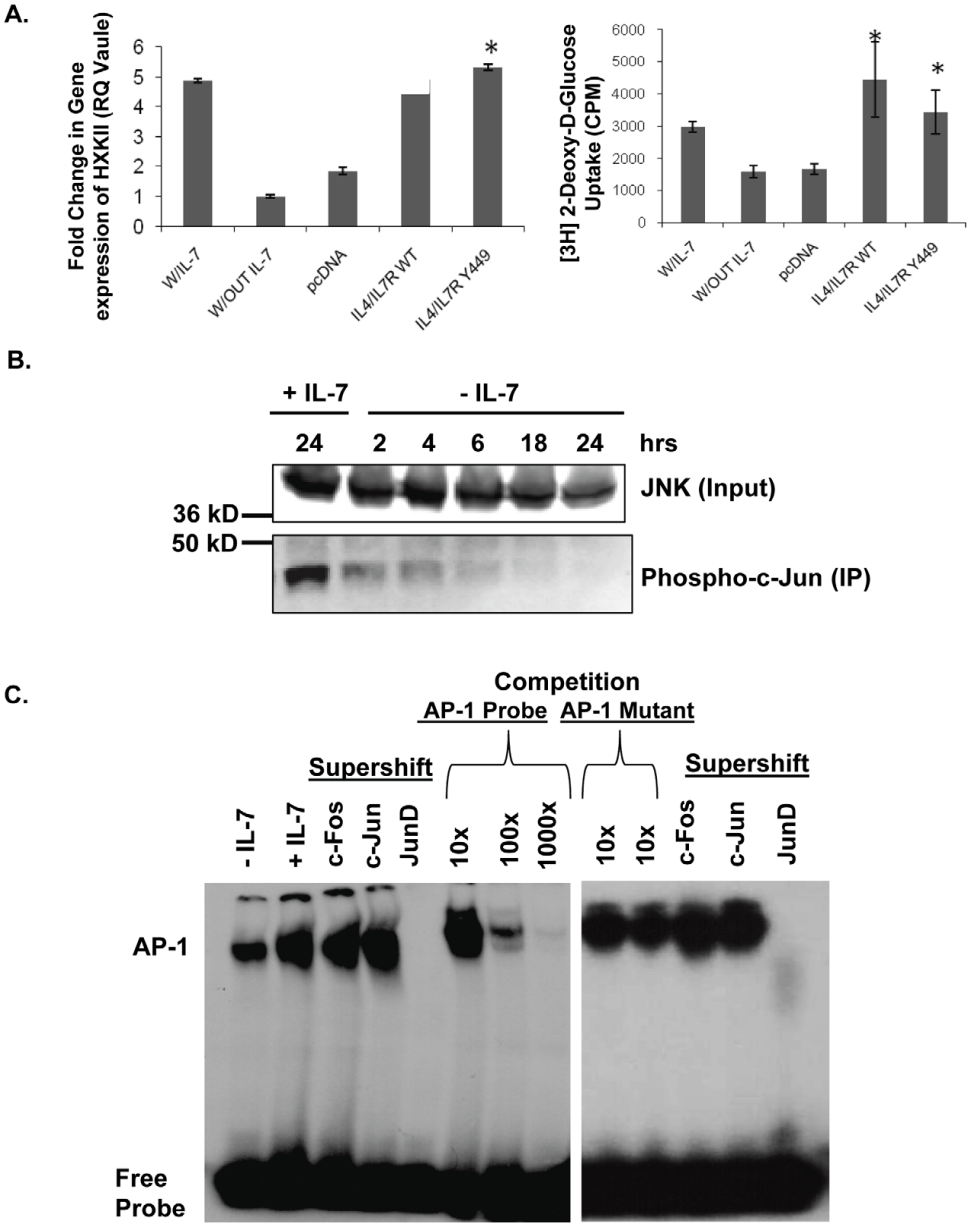
IL-7 signaling induces JNK activity and promotes JunD-containing AP-1 complexes. (A) (Left panel) Quantitative PCR evaluation of HXKII gene expression in the IL-7 dependent T-cell line, D1, after culture with or without IL-7. D1 cells were also nucleofected with a chimeric IL-4/IL-7 receptor (IL-4/IL-7R WT), a chimeric IL-4/IL-7 receptor with a mutation in Y449 (IL4/IL7R Y449) or an empty vector (pcDNA), and stimulated with IL-4, as described in Methods. (Right panel) In cells treated as described above, glucose uptake was assessed by measuring the accumulation of radiolabeled 2-DOG as stated in Methods. (*) indicates a P value of <0.05. (B) To assess JNK kinase activity in response to IL-7, a kinase assay was performed. JNK was immunoprecipitated from whole cell lysates prepared from D1 cells cultured in the presence or absence of IL-7 for the times indicated and the capacity to phosphorylate the kit-supplied substrate, c-Jun, measured as indicator of kinase activity. As input control, pre-immunoprecipitation levels of JNK in lysates are shown. (C) Nuclear lysates prepared from D1 cells grown with or without IL-7 for 18 hours were assayed for AP-1 complex binding to DNA by EMSA, using a radiolabeled DNA probe containing the AP-1 consensus binding site. Supershifts were performed with antibodies specific for c-Fos, c-Jun or Jun-D. Competition was also performed using 10×, 100× or 1000× excess unlabeled AP-1 probe or AP-1 mutant probe. Results (A, B, and C) are representative of three or more independent experiments (values in graphs are mean ± SD).

Previously, using an IL-7 dependent T-cell line, D1, and primary T-cells, we reported that HXKII gene expression was regulated by an IL-7 signal [39]. However, we found that inhibition of STAT5 did not prevent the expression of HXKII in response to IL-7 (Fig. S1). To demonstrate that a STAT5-independent, but still an IL-7 dependent signal, controlled HXKII gene expression, we nucleofected D1 cells with a chimeric IL-4/IL-7 wild-type receptor or with a chimeric IL-4/IL-7 receptor bearing a mutation in the STAT5 binding site, Y449. Normally, when Y449 is phosphorylated it binds STAT5; therefore mutation of this site would prevent STAT5 activation. D1 cells, nucleofected with the chimeric receptors, were cultured with human IL-4. Gene expression of HXKII was measured by quantitative PCR and glucose uptake was measured by 2-DOG uptake. We found that the IL-4-induced gene expression and glucose uptake in nucleofected cells was not affected by the Y449 mutation and that cells were comparable to those incubated with IL-7, suggesting that these events were STAT5-independent (Fig. 1A). Examination of the mouse HXKII promoter region showed that the proximal promoter region contained most of the transcription factors binding motifs that regulate HXKII expression [40]. Some of the recognized factors included SP-1, NF-Y, CREB, Glucose binding site, and the AP-1 complex. To study this, we examined the activity of the MAPK pathway and induction of AP-1 transcription factors in response to IL-7.

### JunD/AP-1 complexes are activated upon IL-7 stimulation through the JNK Pathway

A limited number of studies showed that IL-7 withdrawal up-regulated p38 MAPK [18] and JNK [17], and that ERK signaling was dispensable in IL-7 dependent T-cells [13]. Because of the importance of JNK in AP-1 activation [20], we examined JNK activity in response to IL-7. Previously, we and others established that D1 cells express all the known components of the IL-7 signaling pathway and are representative of the primary T-cell response to IL-7 [19], [36], [37], [39], [41], [42]. Because of this and the fact that primary T-cell subsets are not equally responsive to IL-7 [16], [43], in the current study we used D1 cells, which uniformly respond to the cytokine, to study the functional outcomes of IL-7 signal transduction. Our goal was to identify novel molecular events driving the lymphoproliferative activity of IL-7. A JNK activity assay was performed that involved immunoprecipitation of the kinase from whole cell lysates and detection of the phosphorylated c-Jun substrate as a read out for kinase activity. As shown in the immunoblot for phosphorylated c-Jun substrate in Figure 1B, D1 cells incubated with IL-7 had higher levels of JNK activity than those deprived of IL-7 (Fig. 1B). In IL-7-deprived D1 cells, JNK activity was initially observed through 6 hours of cytokine withdrawal and was undetectable after 18 hours of cytokine withdrawal. As control for protein input, JNK levels were detected in lysates prior to immunoprecipitation. These results support that JNK activation is a part of the IL-7 signaling pathway.

Since JNK is known to regulate the components involved in AP-1 activation, a gel shift assay was performed to determine whether IL-7 induced AP-1 DNA-binding activity. An oligonucleotide probe containing the AP-1 DNA-binding consensus site was incubated with nuclear extracts from D1 cells grown with or without IL-7 for 18 hours. Shown in Figure 1C, EMSA results revealed that more AP-1 bound to DNA in D1 cells incubated with IL-7. This suggested that IL-7 induced the formation of AP-1 complexes that bound to the consensus DNA sequence. To confirm specific binding of the protein to the AP-1 probe, a competition assay with excess cold AP-1 probe (unlabeled) as well as with a mutant AP-1 probe was performed. As seen in Figure 1C, cold AP-1 probe effectively reduced binding of protein to the radiolabeled AP-1 probe, while addition of mutant AP-1 probe had little effect. To identify the components that formed the IL-7-induced AP-1 complex, nuclear extracts, made from D1 cells grown with IL-7, were co-incubated with supershift-specific antibodies against c-Fos, c-Jun and JunD, and added to the AP-1 radiolabeled DNA probe. We found that the c-Fos and c-Jun antibodies did not produce a supershift or impede protein binding to DNA, whereas the JunD antibody caused steric hindrance, preventing protein binding to the AP-1 DNA probe (Fig. 1C). This illustrates that IL-7 stimulation resulted in an AP-1 complex containing JunD.

**Figure 2:**
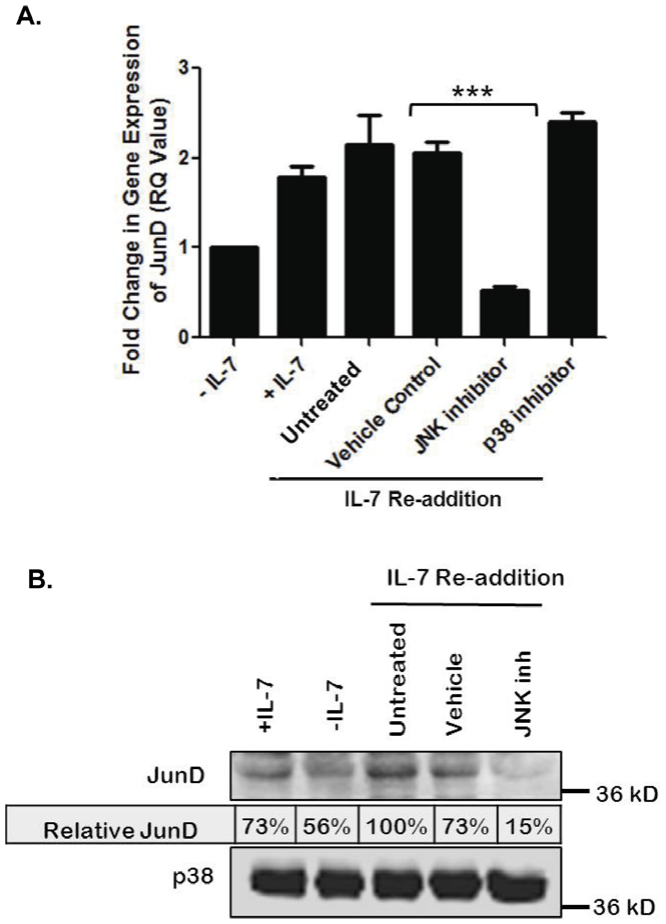
JNK activity promotes JunD expression. (A) Quantitative PCR evaluation of JunD expression in the IL-7 dependent T-cell line, D1. Cells were continuously cultured with IL-7 or without IL-7 for 18 hours, or withdrawn from IL-7 (18 hours) and then re-stimulated for two hours (IL-7 Re-addition). Re-stimulated cells were untreated, treated with DMSO (Vehicle Control), 20 µM JNK inhibitor (JNK inhibitor), or 20 µM p38 MAPK (p38) inhibitor. (***) indicates P<0.0001. (B) Western blot analysis of JunD protein and p38 MAPK protein content, as loading control, of whole cell lysates from D1 cells cultured as stated above. Relative JunD protein indicates JunD protein levels normalized to p38 MAPK (loading control) and compared to the untreated, IL-7 re-addition sample. Results (A, B) are representative of four independent experiments (values in graphs are mean ± SD).

To determine whether the JNK pathway was involved in synthesis and activation of JunD in response to IL-7, we examined, by quantitative PCR, JunD gene expression levels in response to IL-7 and in the presence of JNK (and, as control, p38 MAPK) pharmacological inhibitors. Note that doses of inhibitors used were experimentally determined. D1 cells were grown with or without IL-7 for 18 hours, and, to a separate population of cytokine-deprived cells, IL-7 was added back for 2 hours to induce expression of IL-7-dependent gene products. Loss of IL-7 caused a decrease in the gene expression of JunD that was restored upon a two hour re-addition of IL-7 (Fig. 2A). This IL-7-driven increase in JunD mRNA was inhibited by treatment with the JNK but not the p38 MAPK inhibitor (Fig. 2A). To confirm this result, protein lysates from similarly treated cells revealed that JunD protein decreased in the absence of IL-7 and increased upon IL-7 re-addition (Fig. 2B) in the manner observed for the mRNA levels (Fig. 2A). Inhibition of JNK caused a reduction in protein levels of JunD (Fig. 2B); a result consistent with the gene expression data (Fig. 2A) and the decrease in JNK activity noted after IL-7 withdrawal (Fig. 1B).

### IL-7-dependent JNK activity drives glucose uptake through the synthesis of HXKII

**Figure 3:**
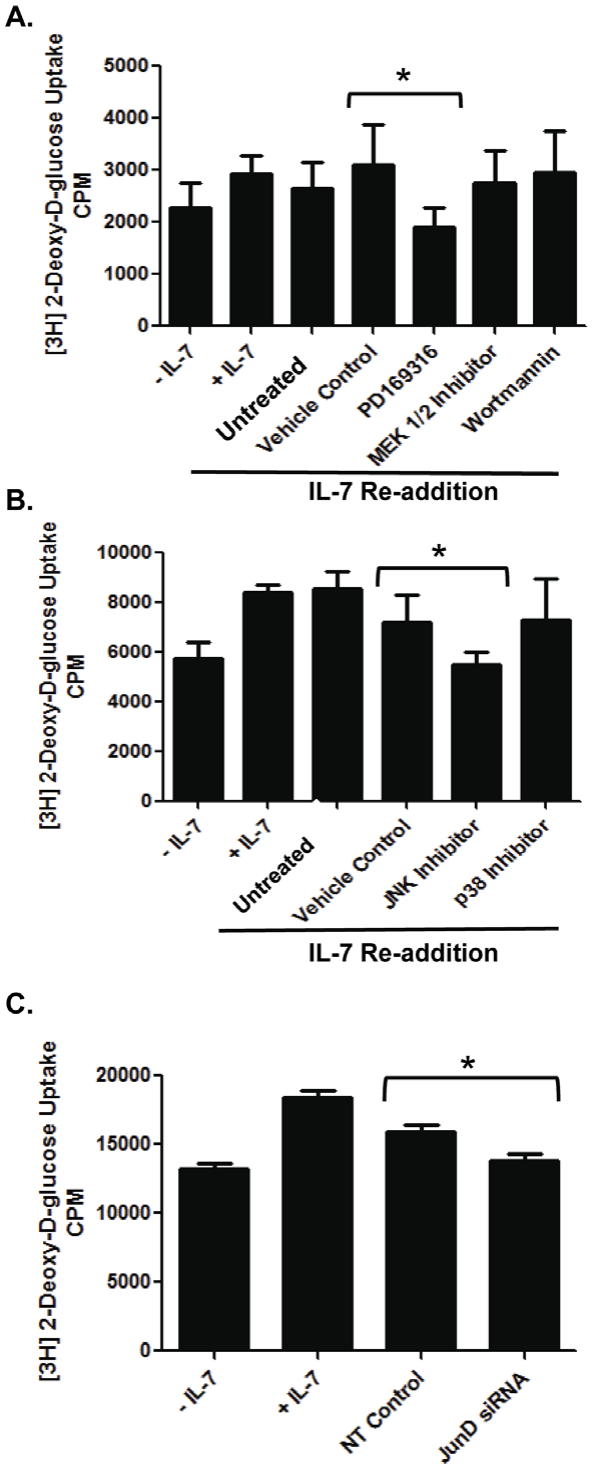
A functional consequence of JNK/JunD signaling is the IL-7 dependent uptake of glucose. (A) D1 cells continuously cultured with or without IL-7 for 18 hours, or withdrawn (18 hours) and then stimulated with IL-7 for four hours (IL-7 Re-addition), were untreated or treated with DMSO (Vehicle Control), 20 µM MAPK inhibitor, 20 µM PD169316, 20 µM MEK1/2 inhibitor or 5 nM PI3K inhibitor, wortmannin. Glucose uptake was assessed by measuring the accumulation of radiolabeled 2-DOG as stated in Methods. (*) indicates P = 0.0348. (B) D1 cells were treated similarly as those in (A) except that cells were pulsed for two hours and specific inhibitors for JNK (20 µM) or p38 MAPK (20 µM) were used. (*) indicates P = 0.0342. (C) D1 cells were cultured with or without IL-7 after introduction of non-specific control (NT) or JunD siRNA as described in Methods. Glucose uptake was assessed as above by measuring the accumulation of radiolabeled 2-DOG. (*) indicates P = 0.0320. Results (A, B, and C) are representative of three or more experiments performed (values in graphs are mean ± SD).

To investigate a functional consequence of JNK activity, D1 cells were deprived of IL-7 for 18 hours and IL-7 added back to the culture with the addition of either PD169316, (p38 MAPK/JNK inhibitor), MEK1/2 inhibitor, or wortmannin (PI3K (Phosphoinositide 3-kinase) inhibitor). As an indicator of cell metabolism, glucose uptake was measured using 2-DOG [39]. Figure 3A showed decreased glucose uptake in D1 cells that were stimulated with IL-7 and incubated with PD169316, an inhibitor of the p38/JNK pathway, but not with inhibitors of the MEK1/ERK pathway or the PI3K pathway. To differentiate between the p38 MAPK and JNK pathways, glucose uptake was then measured in IL-7-stimulated D1 cells cultured with specific inhibitors for JNK or p38 MAPK. Figure 3B demonstrates that D1 cells incubated with a JNK inhibitor, but not a p38 MAPK inhibitor, had decreased glucose uptake upon IL-7 addition. To show that, upon IL-7 stimulation, JunD/AP-1 complexes mediated the uptake of glucose, D1 cells were treated with JunD siRNA and assayed for the uptake of 2-DOG. The efficacy of siRNA knockdown of JunD mRNA (∼60%) and protein (∼50%) are shown in later figures. Inhibition of JunD reduced the uptake of glucose in IL-7-stimulated D1 cells to levels approaching those observed in the cells cultured without IL-7 (Fig. 3C). Note that background levels of 2-DOG uptake are indicated by the IL-7 deprived controls and are the point of comparison for the other experimental samples. These results indicate that JNK and JunD/AP-1 complexes were in part responsible for regulating the activity of factors that promoted glucose uptake upon IL-7 stimulation.

**Figure 4:**
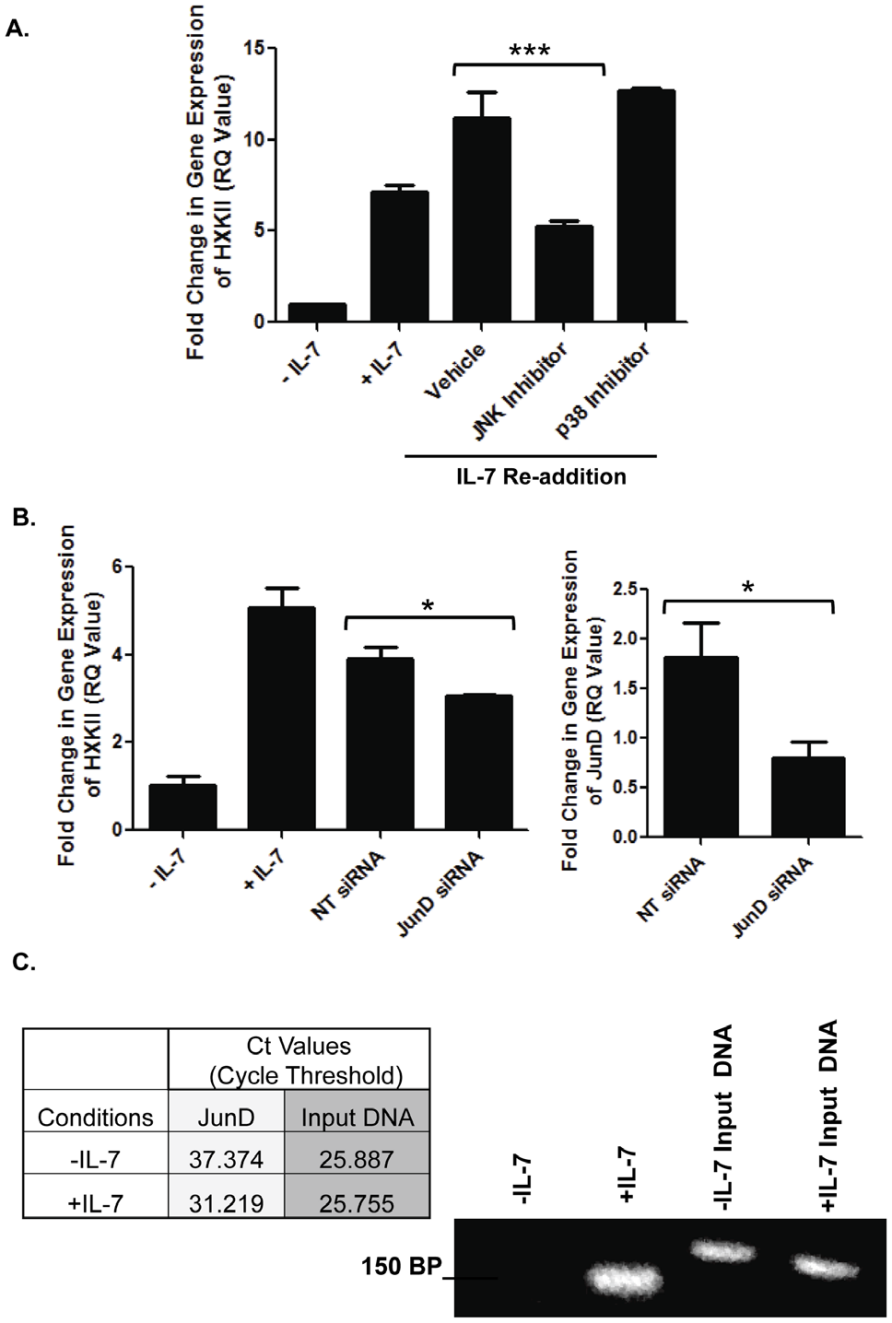
HXKII gene expression is dependent upon JNK/JunD signaling. (A) HXKII gene expression in the IL-7 dependent T cell line, D1, was measured by quantitative PCR as described in Methods. Cells were cultured with or without IL-7, or after an IL-7 pulse for 2 hours (IL-7 Re-Addition), in presence of a vehicle control, 20 µM JNK inhibitor, or 20 µM p38 inhibitor. RQ (Fold change) = 2^−ΔΔCt^. (***) indicates P<0.001. (B) HXKII gene expression in D1 cells cultured with or without IL-7 and the non-targeting control (NT) or JunD siRNA, as described in Methods, was measured as above. (*) indicates P = 0.0254. Efficacy of JunD siRNA upon JunD mRNA levels (right panel) was established through measuring total JunD gene expression by quantitative PCR. (*) indicates P = 0.0336. RQ (Fold change) = 2^−ΔΔCt^. (C) Chromatin Immunoprecipitation (ChIP) was performed using nuclear lysates from D1 cells cultured with or without IL-7 for 18 hours. Results from the quantitative PCR, reported as cycle threshold (Ct) values, are shown in the table. The PCR-amplified 150 bp region of AP-1 promoter DNA from the HXKII gene was visualized by ethidium bromide staining in a non-denaturing agarose gel. Input DNA is shown as equivalent starting materials. Results (A, B and C) are representative of three experiments performed in triplicate (values in graphs are mean ± SD).

In our previous study, we reported that HXKII gene expression increased after 2 hours of IL-7 re-addition to deprived cells [39]. We also found that a STAT5-independent mechanism with driving the synthesis of HXKII (Fig. S1 and Fig. 1A). Since the gene expression of JunD was dependent upon JNK and IL-7 (Fig. 2A), it was possible that the increase of HXKII gene expression that followed IL-7 re-addition was also associated with the activity of JNK. Therefore, the synthesis of HXKII was evaluated in D1 cells stimulated with IL-7 and treated with a JNK inhibitor. Quantitative PCR results demonstrated that HXKII synthesis was reduced by about 60% compared to vehicle control upon treatment with JNK inhibitor (Fig. 4A). D1 cells incubated with the p38 MAPK inhibitor showed little effect (Fig. 4A). This data confirmed that the IL-7-driven increase in HXKII gene expression is likely mediated by JNK. To establish a role for JunD-containing AP-1 complexes in HXKII gene expression, D1 cells were stimulated with IL-7 and treated with JunD siRNA. Shown in Figure 4B is a representative experiment in which loss of JunD reduced the IL-7-driven expression of HXKII. A graph alongside shows that treatment with JunD siRNA reduced JunD mRNA levels by approximately 60% as compared to the non-targeting control siRNA. Hence, JunD/AP-1 complexes were contributing to the synthesis of HXKII in response to IL-7.

The binding of JunD/AP-1 complexes to the AP-1 site on the HXKII promoter was examined to validate our conclusions. To this end, a chromatin immunoprecipitation assay (ChIP) was performed. Nuclear lysates of D1 cells cultured with and without IL-7 for 18 hours were incubated with JunD antibody to immunoprecipitate JunD-bound DNA. Quantitative PCR was then performed, using primers specific to the AP-1 region on the HXKII promoter to amplify a DNA sequence of approximately 150 base pairs. In Figure 4C, we reported the cycle threshold (Ct) values, indicating that amplification of the HXKII promoter DNA, immunoprecipitated with the JunD antibody, from IL-7-cultured cells, occurred 6 cycles faster than from IL-7 deprived cells and was thus significantly more abundant. Endpoint DNA from the amplification reactions was run on a non-denaturing agarose gel to demonstrate that more HXKII promoter DNA was amplified from samples of D1 cells cultured with IL-7. Controls for the reaction are the total DNA inputs, showing equivalent amounts of starting materials. This representative experiment confirms that JunD-containing AP-1 complexes bound to the AP-1 site within the HXKII promoter region in an IL-7 dependent manner.

### Bioinformatics Approach Reveals Novel IL-7 Induced Genes Induced by AP-1/JunD

**Figure 5:**
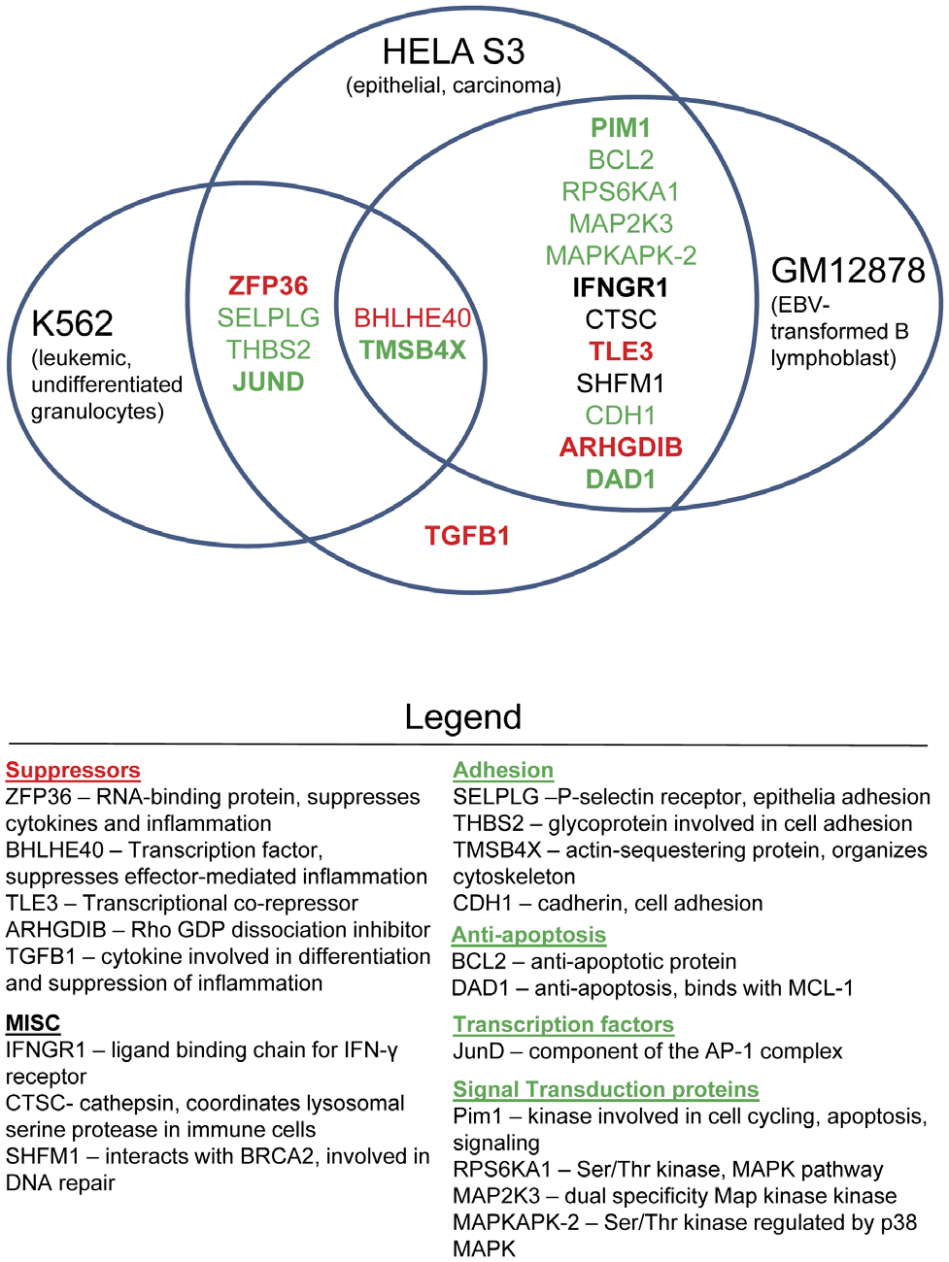
Bioinformatics evaluation of IL-7 dependent gene expression transduced through JunD. Three cell lines (GM12878, Helas3, and K562) were evaluated for genes containing putative JunD binding sites that are potentially IL-7-responsive, as described in Methods. Gene functions were determined using gene ontology analysis as described in Methods, and were separated into suppressive (red) or supportive actions (green).

To identity novel IL-7-regulated genes whose synthesis was driven by JunD/AP-1 complexes, we performed a bioinformatics analysis. Previously, we identified a number of genes whose expression was induced upon IL-7 stimulation or deprivation [31]. A comparison of these genes with potential JunD-targeted genes (identified in a bioinformatics screen, see Methods and Figure S2 with Data S1) yielded a number of targets containing putative JunD binding sites that were potentially IL-7-responsive. The diagram, shown in Figure 5, organizes the information by function. Of the three cell lines characterized, gene functions were separated into suppressive (red) or supportive actions (green). Many of the gene products identified were involved in signal transduction, survival or adhesion (Fig. 5). Among these, JunD emerged as a self-regulated and IL-7 dependent gene (Fig. 5). Suppressive genes were generally involved in blocking inflammatory processes (Fig. 5). The cell line databases assessed for potential JunD target genes were also evaluated for functional homology, whether inhibitory or stimulatory. The most significant gene ontology terms, according to cell line, are listed in order by P-value and are provided as Data S1. Gene ontology ID (GO), significance by p-value, and the number of genes annotated with that term are found in supplemental files. Also included under each cell line are the number of annotated target genes and the number of target genes for the specific cell line. This approach revealed that a variety of functions can be ascribed to JunD-mediated transcription, specifically, regulation of signal transduction, cell cycling and cellular metabolic processes. Of those genes, ones initiated early in the IL-7 signaling cascade, which could drive additional growth pathways, were of interest for study.

### Pim1 is an IL-7-Inducible Gene Product through the Activity of AP-1/JunD

**Figure 6:**
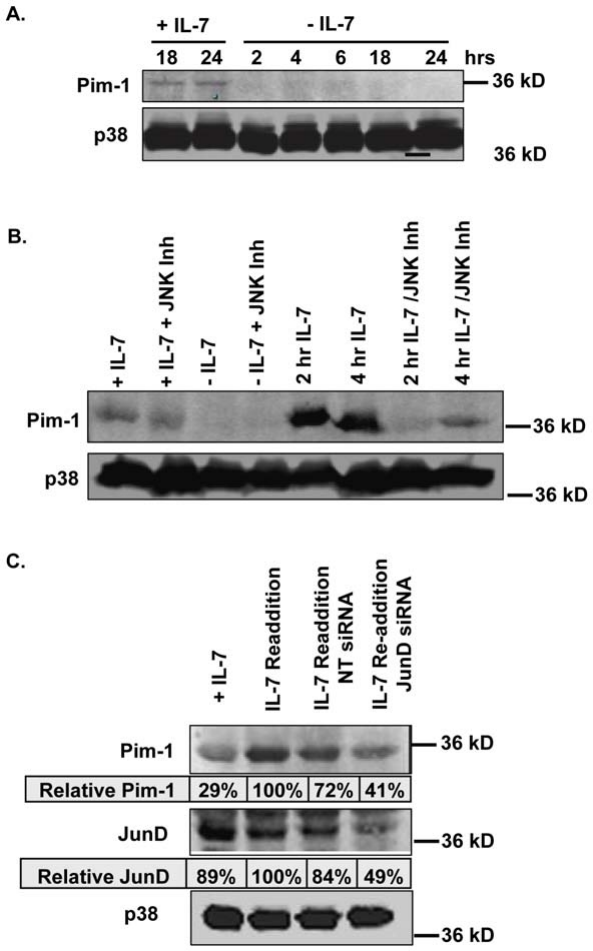
Pim-1 protein is dependent on IL-7 and JNK/JunD. (A) Immunoblot analysis of Pim-1 was performed. Whole cell lysates were prepared from D1 cells cultured in the presence or absence of IL-7 for the indicated time points. p38 MAPK was measured as a loading control. (B) Immunoblot analysis of Pim-1 was performed. Whole cell lysates were prepared from D1 cells cultured in the presence (+) or absence (−) of IL-7 and/or with JNK inhibitor (20 µM), or 2–4 hour IL-7 stimulation after 18 hour deprivation, alone, or with 20 µM JNK inhibitor. p38 MAPK was used as a loading control. (C) Whole cell lysates of D1 cells were cultured for 52 hours with IL-7 in the presence of non-targeting control (NT) or JunD siRNA, then cells were withdrawn from IL-7 for 18 hours as described in Methods. A separate group was also deprived of IL-7, and then re-stimulated with IL-7 for two hours in the presence of siRNA (Re-addition). Whole cell lysates were subjected to SDS-PAGE and immunoblotted for Pim-1, JunD, and p38 as loading control. Tables indicate relative amounts of protein normalized to p38 content and shown relative to the IL-7 re-addition sample. Results are representative of three experiments performed.

One of the genes that emerged from the bioinformatics analysis as a JunD target and IL-7 dependent was the proto-oncogene, Pim-1. Hence, Pim-1 was chosen for evaluation to validate the bioinformatics approach and provide insight on a novel aspect of the IL-7 signal. The repetitive blot in Figure 6A revealed detectable levels of Pim-1 protein in D1 cells grown continuously with IL-7 for 18–24 hours, while withdrawal of IL-7 for 2–24 hours caused a decline in Pim-1. Next, we examined the effect of JNK inhibition upon Pim-1 protein levels in D1 cells grown with or re-stimulated with the cytokine. Our data in Figure 6B indicates that the basal levels of Pim-1 protein expressed in D1 cells, continually growing in IL-7, were only slightly decreased by inhibition of JNK; however, the increased amounts of Pim-1 that were induced upon re-stimulation with IL-7 for 2 or 4 hours after deprivation were almost completely inhibited by blocking JNK activity. These results suggest that basal levels of Pim-1 were in part JNK-dependent, but that induction of Pim-1 protein upon IL-7 re-addition was almost completely dependent upon JNK signaling (Fig. 6B). To establish that JunD/AP-1 factors were transducing the JNK signal that induced Pim1, we repeated the experiment using JunD siRNA and observed a measureable decrease in Pim-1 protein induction after IL-7 re-addition (Fig. 6C) when JunD expression was impaired. Levels of JunD protein in these cells are shown for comparison.

**Figure 7:**
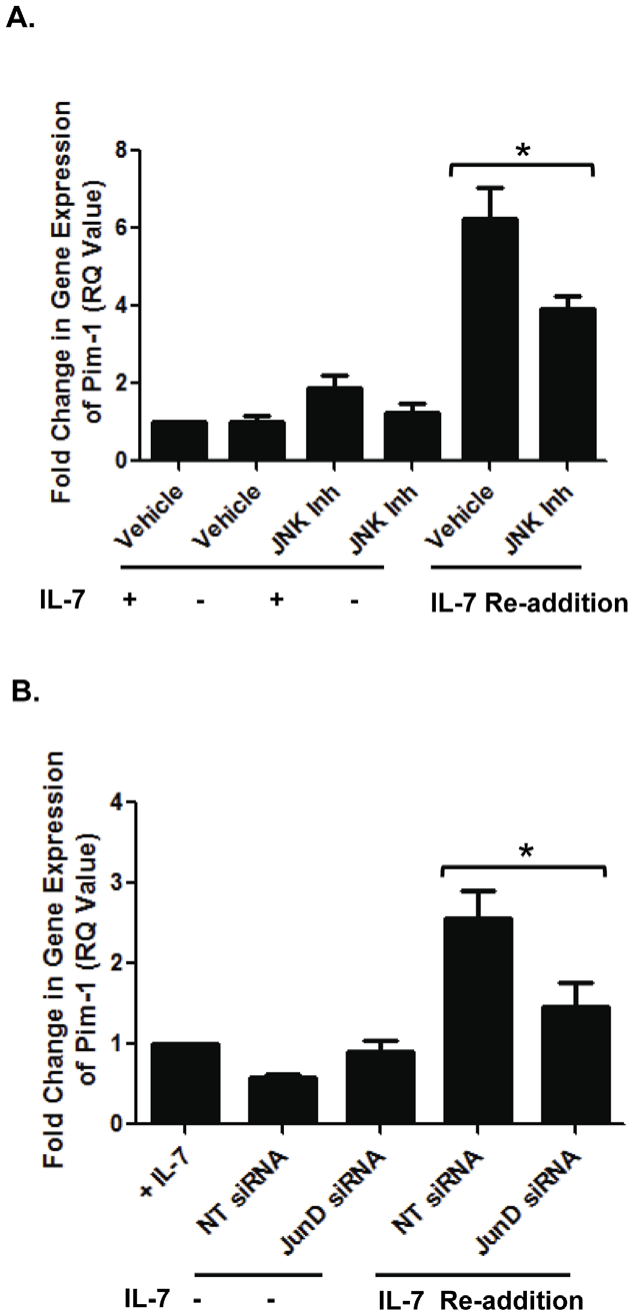
IL-7 inducible Pim-1 gene expression is dependent on JNK/JunD. (A) Pim-1 gene expression in the IL-7 dependent T cell line, D1, was measured by quantitative PCR as described in Methods. D1 cells were cultured with or without IL-7 (+/− IL-7), in the presence of a vehicle control (Vehicle) or 20 µM JNK inhibitor (JNK Inh). In some samples, after an 18 hour deprivation, IL-7 was added back to the culture for 2 hours in the presence of a vehicle control (IL-7 Re-addition, Vehicle) or 20 µM JNK inhibitor (IL-7 Re-addition, JNK Inh). (*) indicates P = 0.0104. (B) Quantitative PCR of Pim-1 gene expression was performed using D1 cells cultured for 52 hours with IL-7 (+) and then 18 hours without IL-7 (−) in the presence of non-targeting control (NT) or JunD siRNA. A separate group was also deprived of IL-7, and then re-stimulated with IL-7 for two hours in the presence of siRNA (IL-7 Re-addition). RQ (Fold change in gene expression normalized to β-actin) = 2^−ΔΔCt^. Results are representative of three experiments performed in triplicate (values in graphs are mean ± SD). (*) indicates P = 0.0140.

These studies were followed by examining Pim-1 gene expression in response to either JNK or AP-1/JunD inhibition. In Figure 7A, re-addition of IL-7 caused an increase in Pim1 mRNA supporting that increase previously detected by protein assay (Fig. 6B). Inhibition of JNK decreased the levels of Pim-1 mRNA in D1 cells stimulated with IL-7, while little or no effect was observed in D1 cells continually grown in IL-7. Likewise, as observed for protein levels of Pim-1, inhibition of JunD with siRNA decreased Pim-1 gene expression in cells re-stimulated with IL-7 after deprivation (Fig. 7B).

### Proliferation of Primary Lymphocytes in Response to IL-7 Depends on JunD Activity

**Figure 8:**
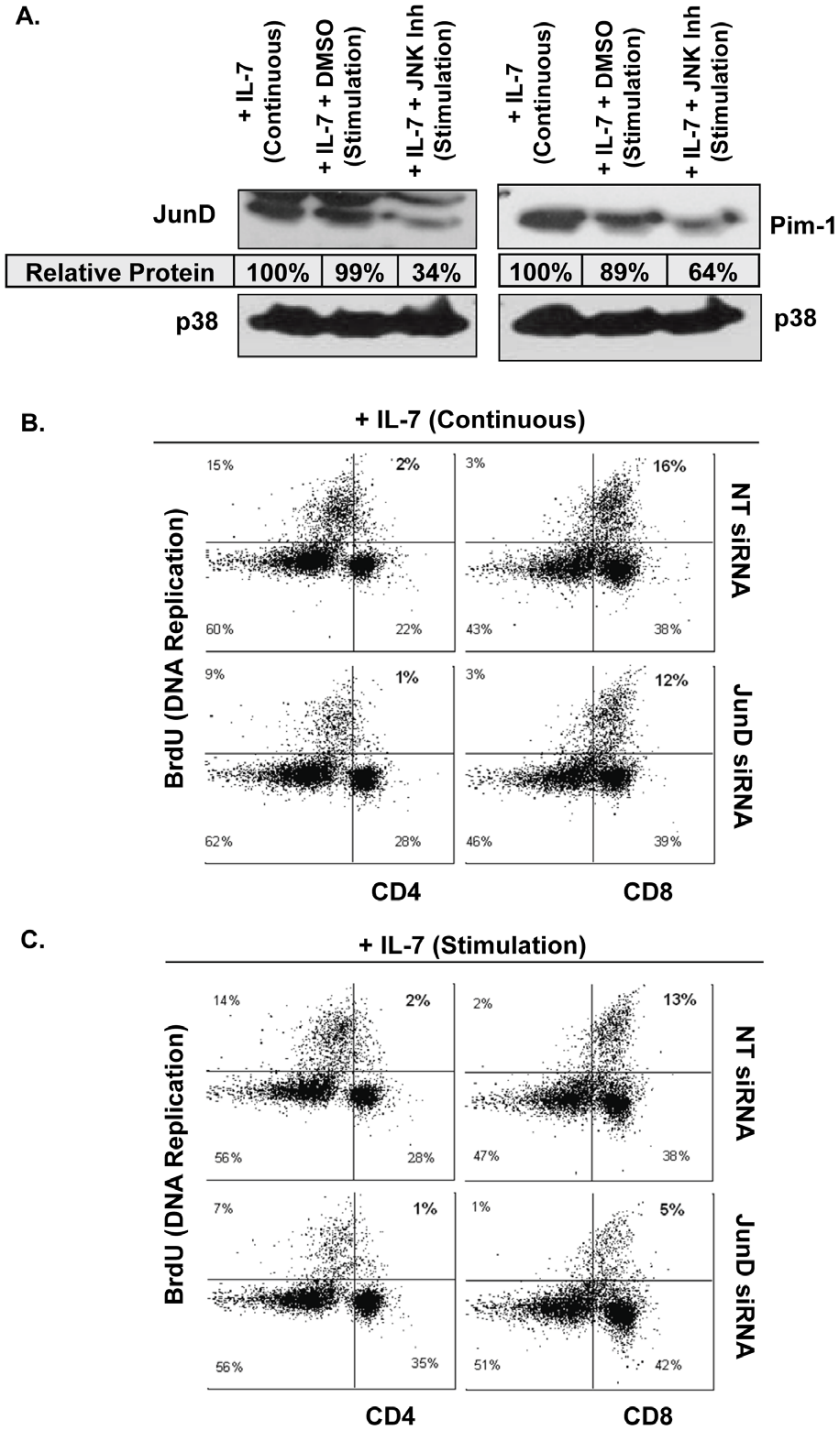
Inhibition of JunD Prevents IL-7 Induced Proliferation of Primary Lymphocytes. (A) Lymph node T-cells were isolated from WT C57Bl/6 mice, cultured continuously with 150 ng/ml of IL-7 for 7 days (Continuous) or for 5 days, then deprived from IL-7 overnight, and IL-7 re-added for 24 hours (Stimulation) and treated with vehicle (DMSO) or 20 µM JNK inhibitor as described in Methods. Whole cell lysates were prepared from T-cells, subjected to SDS-PAGE and immunoblotted for Pim-1, JunD, and p38 MAPK as loading control. Tables indicate amounts of protein normalized to p38 MAPK content and shown relative to the IL-7 (Continuous) sample. Results are representative of two experiments performed. (B, C) Proliferation was measured by BrdU incorporation. Lymph node T cells were isolated from WT C57Bl/6 mice as described in (A) and treated with non-targeting control (NT) or JunD siRNA as described in Methods. Cells were assessed for BrdU incorporation and surface expression of CD4 and CD8 as determined by flow cytometric analysis of BrdU-PE and CD4- or CD8-PerCp fluorescence. Dot blots show percentages representing the population of cells that are non-proliferating (BrdU negative), proliferating (BrdU positive), and CD4+ or CD8+ as indicated by the quadrants. Quadrants were established using controls. Gating was performed to remove autofluorescent cells. Results are representative of duplicate samples.

Our studies with the D1 cell line revealed the specifics of the IL-7 signaling pathway from activation of JNK through induction of the JunD/AP-1 transcription factors and increased gene expression of HXKII and Pim-1. The expected outcome of IL-7 signal transduction through this pathway would be T-cell proliferation. To examine this, we used our *ex vivo* expansion protocol [43] to isolate IL-7-dependent primary T-cells. This expansion method is needed because freshly isolated lymphocytes contain only a small number of T-cells that proliferate in response to IL-7 (∼10–20%, Khaled, unpublished data). To confirm the results observed with D1 cells, that JunD and Pim1 expression was dependent on the JNK pathway, we treated IL-7 stimulated T-cells with a JNK inhibitor and assessed the effect upon protein levels of JunD and Pim-1. Results in Figure 8A show that JNK inhibition resulted in decreased JunD and Pim-1 proteins (34% and 64% respectively), substantiating the data from D1 cells. Note that primary T-cells under IL-7-deprived conditions cannot be evaluated because these fail to expand and die in culture. To evaluate IL-7-driven proliferation, we measured the incorporation of BrdU in CD4 and CD8 T-cells. As shown in Figure 8B, continuous culture with IL-7 caused CD8 T-cell expansion (16%) to occur more rapidly as compared to CD4 T-cells (2%) – an observation previously published [16]. The addition of JunD siRNA had a modest inhibitory effect (Fig. 8B). However, examining the proliferation of CD4 and CD8 T-cells, re-stimulated with IL-7, revealed that JunD inhibition reduced IL-7 driven growth by more than 60% - more so for the actively dividing CD8 T-cells (from 13% to 5%) (Fig. 8C). These results suggest that IL-7 signaling promotes T-cell proliferation in part through JunD-mediated transcription of gene products such as Pim-1 or HXKII.

In summary, we have shown that IL-7 promotes growth signals through other transcriptional components in addition to STAT5. We found that the JunD/AP-1 transcription factors are activated in response to JNK and drive the synthesis of at least two essential mediators of lymphocyte growth, HXKII and Pim-1, in response to IL-7 stimulation. These results establish a novel approach employing bioinformatics to discover new transducers of the IL-7 metabolic and proliferative signals.

## Discussion

Our results suggest that IL-7 in part contributes to the metabolism and growth of T-cells by promoting HXKII and Pim-1 gene expression through the JNK activation of JunD/AP-1. This conclusion was reached by finding that JNK was active in response to IL-7, and that IL-7 induced the binding of AP-1 transcription factors, containing JunD, to DNA sequences such as the HXKII promoter. Using a bioinformatics approach to discover previously unrecognized JunD gene targets that were IL-7-responsive, we identified Pim-1. Confirmation that Pim-1 expression was IL-7- and JunD-dependent, validated the bioinformatics results. Physiological relevance of our findings was established when inhibition of JNK in primary lymphocytes decreased the levels of Pim-1 and JunD and inhibition of JunD prevented the proliferation of CD8 T-cells stimulated with IL-7. This work achieves the goal of demonstrating that other regulators of gene expression, in addition to the JAK/STAT5 pathway, can respond to the IL-7 signal in T-cells and indicates that a bioinformatics approach can be used to discover novel IL-7 signal transducers.

To support T-cell growth, IL-7 signaling leads to the activation of transcription factors, such as STAT5. STAT5 can mediate T-cell survival by inducing anti-apoptotic proteins of Bcl-2 family [44], [45]. STAT5 also promotes glucose metabolism by enabling GLUT1 trafficking to the cell surface [38]. However, we found that the IL-7-mediated increase of HXKII gene expression used a STAT5 independent mechanism to regulate glucose uptake in T-cells. We identified JunD-containing AP-1 complexes as key factors that controlled the synthesis of HXKII in response to IL-7. Pim-1 is also a recognized gene transcribed by STAT5 [46] and a possible JunD-target. We found that T-cells deprived and then re-stimulated with IL-7, which mimics the *in vivo* conditions in which T-cells encounter IL-7, immediately increased Pim-1 levels in a JunD/AP-1 dependent manner. This is consistent with the proliferative function of Pim-1, since it phosphorylates and induces the activity of proteins involved in cell cycling, including Cdc25A [47] and Skp2 [48]. Our conclusions are supported by the fact that Pim-1 is an effector of the IL-7 signaling pathway, as its expression reconstituted thymic cellularity in IL-7 deficient mice [49], and that Pim-1, 2 and 3 are required for proliferation of peripheral T-cells [50].

JunD was initially described as a negative regulator of proliferation. Early studies showed that over expression of JunD in immortalized fibroblasts increased the numbers of cells in the G0/G1 phase of the cell cycle, promoting growth arrest [30]. JunD also suppressed transformation by Ras [30]. Immortalized cells lacking JunD had higher levels of cyclins and increased proliferation, but also were more sensitive to death-inducing agents, suggesting that JunD could have both negative and positive effects upon cell growth [51], [52]. Concerning the latter activity, JNK and JunD were shown to work with NF-kB to increase the expression of the caspase inhibitor, cIAP-2 [52]. While these studies were performed using immortalized cells and fibroblasts, the evaluation of JunD activity in lymphocytes revealed different results and demonstrated that JunD activity can be cell specific. JunD over expression did not protect lymphocytes from apoptosis and caused reduced growth and activation, while JunD^−/−^ T-cells hyperproliferated upon stimulation [53]. These findings seemed in apparent conflict with our data that HXKII, a key enzyme in the glycolytic pathway, was a JunD target gene. To explain this, we performed a bioinformatics evaluation of potential JunD-regulated genes and found that many critical effectors of cell signaling and growth (like Pim-1), survival (like Bcl-2), and metabolism were possible JunD targets. Further, we found that inhibition of JunD impaired the proliferation of T-cells in response to IL-7. While, JunD remains a negative regulator of growth in many cells, like intestinal epithelial cells in which JunD repressed gene expression of cyclin dependent kinase 4 (CDK4) [54], in IL-7-pulsed T-cells, as shown in our studies, JunD promotes the expression of essential growth effectors.

Regulation of JunD gene expression is not typical of other AP-1 components. Contrary to results with other AP-1 proteins, JunD protein levels were detected in quiescent immortalized cells; JunD protein then initially declined upon serum stimulation and later steadily increased [30]. Coupled to the constitutive activity of the JunD promoter, this suggests that JunD may be controlled by different post-transcriptional or post-translational mechanisms. As example, the JunD transcript is intronless, is G/C rich, has a long 5′ untranslated region (UTR) and produces two isoforms, a full-length and truncated form (reviewed in [55]). Moreover, the presence of a unique post-transcriptional control element (PCE) and the potential interaction with RNA helicase A (RHA) suggests that JunD could be efficiently translated under the right growth conditions [56]. Post-translational modifications also contribute to the activity of JunD in different cellular environments. JunD is poorly ubiquitinated and has a long half-life [57]. The JunD transcript is also positively regulated by JNK activity [58], as we have shown in the IL-7-dependent D1 cells. In fact, JNK could stimulate an auto-regulatory loop that controls the gene expression of JunD.

In summary, JunD emerges as an important transducer of the IL-7 signal in T-cells and, along with the JAK/STAT pathway, could promote gene expression to drive survival and growth when cells are stimulated with IL-7. The importance of this conclusion is appreciated when considering that T-cells normally exist in an IL-7-limited environment (with picograms levels of the cytokine normally detected in serum [59]). To be stimulated, T-cells must traffic to IL-7-containing tissues. When a T-cell receives an lL-7 signal, the JNK/JunD pathway may function to enhance the JAK/STAT mechanism and induce expression of genes like HXKII and Pim-1 as a rapid response to the initial growth signal. Because the therapeutic use of IL-7 involves application of superphysiological dosing of the cytokine that is very different from the *in vivo* state of limited IL-7 availability, there is a need to better understand the consequences of IL-7 signal transduction and the impact upon gene expression in T-cells. The fact that the JNK/JunD pathway can have positive outcomes in IL-7 dependent T-cells highlights the need for continued research in this area. The bioinformatics approach presented could provide a means to identify potential gene expression changes that result from activation of JunD/AP-1 complexes in the context of IL-7 signaling that can be tested for validation in different animal models of immunological responses.

## Materials and Methods

### Mice, Cell Lines and Culture Reagents

C57Bl/6 mice were purchased from Jackson Laboratory (Bar Harbor, Maine) and housed at the University of Central Florida, Orlando, FL. Mice were used in accordance with the recommendations in the Guide for the Care and Use of Laboratory Animals of the National Institutes of Health. The protocol was approved by the Institutional Animal Care and Use Committee at the University of Central Florida (Assurance # A4135-01). All efforts were made to minimize suffering. The IL-7 dependent T cell line, D1, was established from pro-T lymphocytes isolated from a p53^−/−^ mouse as previously described [31]. Since its establishment in 1997, the D1 cell line has been used to study regulatory pathways controlled by IL-7 [19], [33], [36], [39], [60]. D1 T-cells were grown in RPMI 1640, 10% fetal bovine serum (FBS), 5% Penicillin/Streptomyocin (Fisher), 0.1% B-mercaptoethanol (Invitrogen) (complete medium) and 50 ng/mL IL-7 (Peprotech). Early passages of D1 cells were frozen as stocks and cells used at less than 10 passages from stocks. Primary lymph node (LN) T cells were isolated from 8- to 12-week-old C57Bl/6 mice as previously described [43]. To enrich for IL-7 dependent cells, we used our published method of *ex vivo* expansion, culturing LN T cells with 150 ng/mL IL-7 for 5 days [43]. Reagents used as described in Figure Legends include: JNK inhibitor II (Calbiochem, 20 µM), p38 MAPK inhibitor (Calbiochem, 20 µM), Wortmannin (Calbiochem, 5 nM), PD169316 (Calbiochem, 20 µM), MEK1/2 inhibitor (Calbiochem, 20 µM) and STAT5 inhibitor (Calbiochem, 50 µM).

### Plasmids and Nucleofection for Transient Gene Expression

Generation of the chimeric IL-4 receptor/IL-7 receptor plasmids were previously described [60] and pcDNASTAT5a-CA was a kind gift from Dr. Wenqing Li, NCI-Frederick. To transiently express the plasmids, T-cells were “nucleofected” using the Murine T-cell Nucleofection kit (Amaxa), following the manufacturer's protocol. Briefly, 1×10^6^ D1 cells were incubated with (4 µg) plasmid DNA in 100 µl of the mouse T-cell solution, and electroporated with the specific program optimized for mouse T-cells. Nucleofected D1 cells were incubated in the supplemented media with or without IL-7 for 4–8 hours prior to analysis. In D1 cells nucleofection efficiency averaged approximately 40–50% expression of the target gene with viabilities ranging 60–80%.

### Glucose Uptake Assay

Cells were incubated in glucose-free, serum-free RPMI 1640 supplemented with or without IL-7 for 1 hour. 2-Deoxy-D-[_3_H] glucose (2-DOG)(2 µCi/reaction) (Sigma) was added for 3 min. Reactions were stopped by adding 250 µl of ice-cold 0.3 mM phloretin (Sigma). Cells were then centrifuged through a cushion of 10% bovine serum albumin, and lysed with 0.1% Triton X-100. Radioactivity was measured with scintillation counter (LS6500, Beckman Coulter).

### Real Time PCR

Ten million cells per experimental condition were re-suspended in 1 ml of TRIzol reagent (Invitrogen). Total RNA was extracted from the cells using the reagent according to the manufacturer's instructions. Each cDNA template was synthesized from total RNA by reverse transcription with iScript cDNA Synthesis kit (BioRad) according to manufacturer's instructions. Quantitative analysis of cDNA amplification was assessed by incorporation of SYBR Green (ABI) into double-stranded DNA. Primer sets were used as follows: for mouse Pim-1: 5′-CCC GAGCTATTGAAGTCTGA-3′, 5′-CTGTGCAGATGGATCTCA GA-3′ (sense and antisense, respectively) [61], for mouse JunD: 5′-ATGGACACGCAGGAGCGCAT-3′ and 5′-AGCAGCTGGCAGCCGCTGTT-3′ (sense and antisense, respectively [62], for mouse HXKII: 5′-CACTGGGTACTAAGGCTCAA-3′ and 3′-CGGAGTTGTTCTGCTTTGGA-5′ [39], and for β-Actin: 5′-GAAATCGTGCGTGACATCAA AG-3′ and 5′-TGTAGTTTCATGGATGCCACAG-3′ (sense and antisense, respectively) [63]. Reactions contained Fast SYBR Green Master mix (1×), β-Actin primers (50 nM), or Pim-1 or JunD primers (100 nM), and 3–4 µg cDNA template. Thermal cycling conditions were as follows: enzyme activation for 20 seconds at 95°C, followed by 40 cycles of 3 seconds at 95°C, and 30 seconds at 62°C at annealing and extension temperatures. All cDNA samples were processed using the ABI Fast 7500 and analyzed using ABI Sequence Detection Software version 1.4. The difference in mRNA expression was calculated as follows: fold change = 2^−ΔΔCt^, ΔΔCt is equal to the change in ΔCt values over time after normalization to β-actin.

### Detection of Proteins by Immunoblotting

For preparation of whole cell lysates, 20–25×10^6^ D1 cells (per experimental sample) were lysed using the Cell Lysis buffer (Cell Signaling) in the presence of protease and phosphatase inhibitors (Roche). Lysates were immunoblotted for Pim-1 and JunD as described below. For immunoblotting, whole cell lysate samples were run in 12% SDS-PAGE gels, and proteins transferred to nitrocellulose membranes (Invitrogen) following manufacturers' protocols. Membranes were washed and probed with primary antibodies (see below) and incubated with horseradish peroxidase (HRP)-conjugated (Santa Cruz) or fluorescence-conjugated secondary antibodies (LICOR). Signal was detected using either chemiluminescent substrate (SuperSignal West Fempto; ThermoSci) or the LICOR Odyssey detection system. The primary antibodies used in this study were as follows: a mouse monoclonal antibody against Pim-1 (Abcam), and rabbit polyclonal antibodies against p38 (Santa Cruz Biotechnology), and JunD (Abcam).

### JunD Inhibition by Small Interfering RNAs (siRNAs)

Four million D1 cells (per experimental condition) were treated with JUND1 SMART pool siRNA (Dharmacon) and Accell delivery media (Dharmacon) supplemented with 1% FBS, and IL-7 (50 ng/ml) for 52 hours. After 52 hours, cells were washed and re-plated in media containing the siRNA alone, for 18 hours with or without IL-7. In addition, a subset of IL-7 deprived cells was re-pulsed with the cytokine. SMART pool siRNA contains four sequence variations of siRNAs to eliminate non-specific interactions. Non-targeting siRNA (NT siRNA) and NT siRNA, containing a FAM reporter, were used as controls and to determine delivery efficiency, respectively.

### Detection of JNK Activity

Whole cell lysates from 10×10^6^ D1 cells (per experimental sample) were prepared using the Kinase Extraction Buffer provided within the JNK kinase assay kit (KinaseSTAR JNK Activity Assay Kit, BioVision) and assayed for protein concentration by absorbance at 280 nm (Nanodrop 8000). To measure JNK activity, cell lysates were pre-cleared with Protein A/G Sepharose beads (Santa Cruz) and then incubated with the kit provided JNK-specific antibody and agarose beads to pull down JNK. Phospho-c-Jun was used as a substrate to measure JNK activity following the manufacturer's protocol. Total JNK protein in lysates was measured using a JNK-specific antibody (Cell Signaling) following the immunoblot procedure described above.

### Chromatin Immunoprecipitation (ChIP) Assay

ChIP assay was performed using the ChIP-IT kit (Active Motif) according to manufacturer's protocol. Briefly, 100×10^6^ D1 cells were incubated overnight with or without IL-7, and treated with 37% formaldehyde to crosslink protein to DNA. Cells were then treated with the kit-provided lysis buffer supplemented with protease inhibitors and dounce homogenized. Nuclear pellets were sonicated and pre-cleared using the Protein G beads provided within the kit. Pre-cleared chromatin was incubated with anti-JunD antibody (Abcam) and immunoprecipitated using Protein G beads according to the manufacturer's protocol. DNA was eluted and purified. To determine the DNA sequence to which JunD bound, PCR analysis of the DNA was performed using AP-1 primers for the HXKII promoter sequence (Primer3 software): forward Primer 5′GGGCTCTAGGCGCTGATT3′ and reverse Primer: 5′GGAGTTGGTGCAACAATGTG3′. PCR products were analyzed by non-denaturing agarose gel (1%) electrophoresis.

### Electrophoretic Mobility Shift Assay (EMSA)

Nuclear extraction and EMSA were performed according to the method described by Jaganathan et al. [64] and a modified Dignam protocol [65]. Briefly, 60×10^6^ cells were washed and cell lysates prepared with hypotonic buffer (20 mM HEPES (pH 7.9), 1 mM EDTA, 1 mM EGTA, 1 mM Na3VO4, 1 mM Na4P2O7, 0.5 mM PMSF, 0.1 mM aprotinin, 1 mM leupeptin, 1 mM antipain, and 1 mM DTT). NP-40 was added to a final concentration of 0.2% and the solution was centrifuged. The supernatants were retained as cytosolic controls and the nuclear pellets lysed with hypertonic extraction buffer (20 mM HEPES (pH 7.9), 0.42 M NaCl, 1 mM EDTA, 1 mM EGTA, 1 mM DTT, 20 mM NaF, 20% glycerol, 1 mM Na3VO4, 1 mM Na4P2O7, 0.5 mM PMSF, 0.1 mM aprotinin, 1 mM leupeptin, 1 mM antipain). The nuclear fractions were then recovered by centrifugation. Transcription factors bound to specific DNA sequences were examined by EMSA. Normalized extracts, containing 3–8 µg of total protein, were incubated with a double-stranded ^32^P-radiolabeled AP-1 oligonucleotide probe (Santa Cruz) prepared by radiolabeling the AP-1 probe with [α^32^P]dCTP (3000 Ci/mmol) and [α^32^P]dATP (3000 Ci/mmol). Protein-DNA complexes were resolved by non-denaturing polyacrylamide gel electrophoresis (PAGE) and detected by autoradiography. To establish the specificity of the AP-1 probe, unlabeled AP-1 oligonucleotides or mutant AP-1 nucleotides (Santa Cruz) were incubated with nuclear extracts for 15 min before incubation with radiolabeled probes. To identify components of the AP-1 transcription factor in the DNA–protein complex shown by EMSA, we used protein-specific supershift antibodies, c-Fos, c-Jun and JunD (Active Motif), to detect the formation of a supershift DNA–protein complex. These antibodies were incubated with the nuclear extracts for 30 min at room temperature before incubation with radiolabeled probe.

### Analysis of JunD ChIP-seq Data

The JunD ChIP-seq (chromatin immunoprecipitation followed by high throughput parallel sequencing) data for three cell lines, GM12878, Helas3, and K562, was downloaded from http://hgdownload.cse.ucsc.edu/goldenPath/hg18/encode DCC/wgEncodeYaleChIPseq/. Narrow peaks provided by this website were used as the binding regions of JunD. These narrow peaks are regions enriched with JunD binding segments in ChIP-seq experiments, and are selected with a false discovery rate [66] of 0.001. In total, 12958, 45893, and 1500 JunD binding regions were obtained from the GM12878, Helas3, and K562 cell lines, respectively. These JunD binding regions were compared with all annotated refseq genes in the human genome. By assuming that the genes closest to the above binding regions were target genes of JunD, we obtained 6754, 15577, and 1643 target genes in the above three cell lines, respectively. For example, we obtained 1643 target genes in the K562 cell line with only 1500 binding regions because some binding regions were located in multiple annotated genes. To understand the function of these target genes, we performed gene ontology analysis by using the GOTermFinder software [67]. Results are found in supplemental files, Figure S2 and Data S1. A previous cDNA array, using IL-7 dependent D1 cells [31], identified 179 genes that were immediate responders to a two hour pulse with IL-7. We found that 116 out of the 179 IL-7-responsive genes were target genes of JunD in the three cell lines. However, 108 out of the 116 genes are not annotated.

### Proliferation Assay for Primary Lymphocytes

Five to ten million primary LN T-cells, enriched by 5 day culture with IL-7 and treated with control or JunD siRNA as described above, were examined for proliferative capacity. Viability of cultured cells was determined based on cell shrinkage and granularity by gating on FSC/SSC parameters acquired by flow cytometry (Accuri C6 Flow Cytometer). IL-7-cultured primary T-cells treated with siRNAs were pulsed with 10 µM BrdU for 48 hours. DNA synthesis was evaluated through BrdU incorporation, using a PE-tagged, anti-BrdU antibody for detection of DNA content with a commercially available kit according to manufacturer's protocol (BD Biosciences). Surface expression of CD4 and CD8 on the primary T cells were assessed by flow cytometry using the following conjugated antibodies: PerCP-anti-CD4 (clone RM4-5), and PerCP-anti-CD8 (clone 53-6.7) (BD Biosciences). Cells were incubated with antibodies for 20 min on ice, washed in RPMI+10% FBS, prior to BrdU analysis, and analyzed by flow cytometry on a C6 flow cytometer (Accuri).

### Statistics

Statistical analysis and significance was determined using Prism 5 (Graphpad) for Windows, Version 5.02.

## Supporting Information

Figure S1 **STAT5 does not promote HXKII gene expression or glucose uptake in response to IL-7.** (A, B) D1 cells were nucleofected with pcDNA (empty vector) or constitutively active STAT5a-CA (A) and evaluated for HXKII gene expression using quantitative PCR or (B) glucose uptake as described in Methods. Results are representative of two independent experiments performed in triplicates (values in graphs are mean ± SD). (C) Quantitative PCR evaluation of HXKII gene expression in D1 cells after culture with or without IL-7 for 18 hours, or with IL-7 in the presence of a STAT5 inhibitor (50 µM) for 18 hours. RQ (Fold change in gene expression normalized to β-actin) = 2^−ΔΔCt^. (*) indicates P value of <0.05.
(TIF)

Figure S2 **Identified JunD target genes in the three cell lines.** JunD-regulated gene targets were identified by ChIP-seq as described in Methods. The top three most significant gene ontology terms that are at least at level 5 for each group of target genes are provided when they are available. For each term, a gene ontology term ID is provided, followed by the p-value of significance, and the number of target genes annotated with the term. The two numbers under each cell line name are the number of annotated target genes and the number of target genes for the specific cell line.
(TIF)

Data S1 **The list of target genes in the three cell lines and their shared function shown in Figure S2 are provided in the supplemental file.**
(XLS)
