# Supplementary figures and images for: JunD/AP-1-Mediated Gene Expression Promotes Lymphocyte Growth Dependent on Interleukin-7 Signal Transduction
Source: PLoS One. 2012 Feb 23;7(2):e32262. doi: 10.1371/journal.pone.0032262 (PMC3285677; doi:10.1371/journal.pone.0032262)

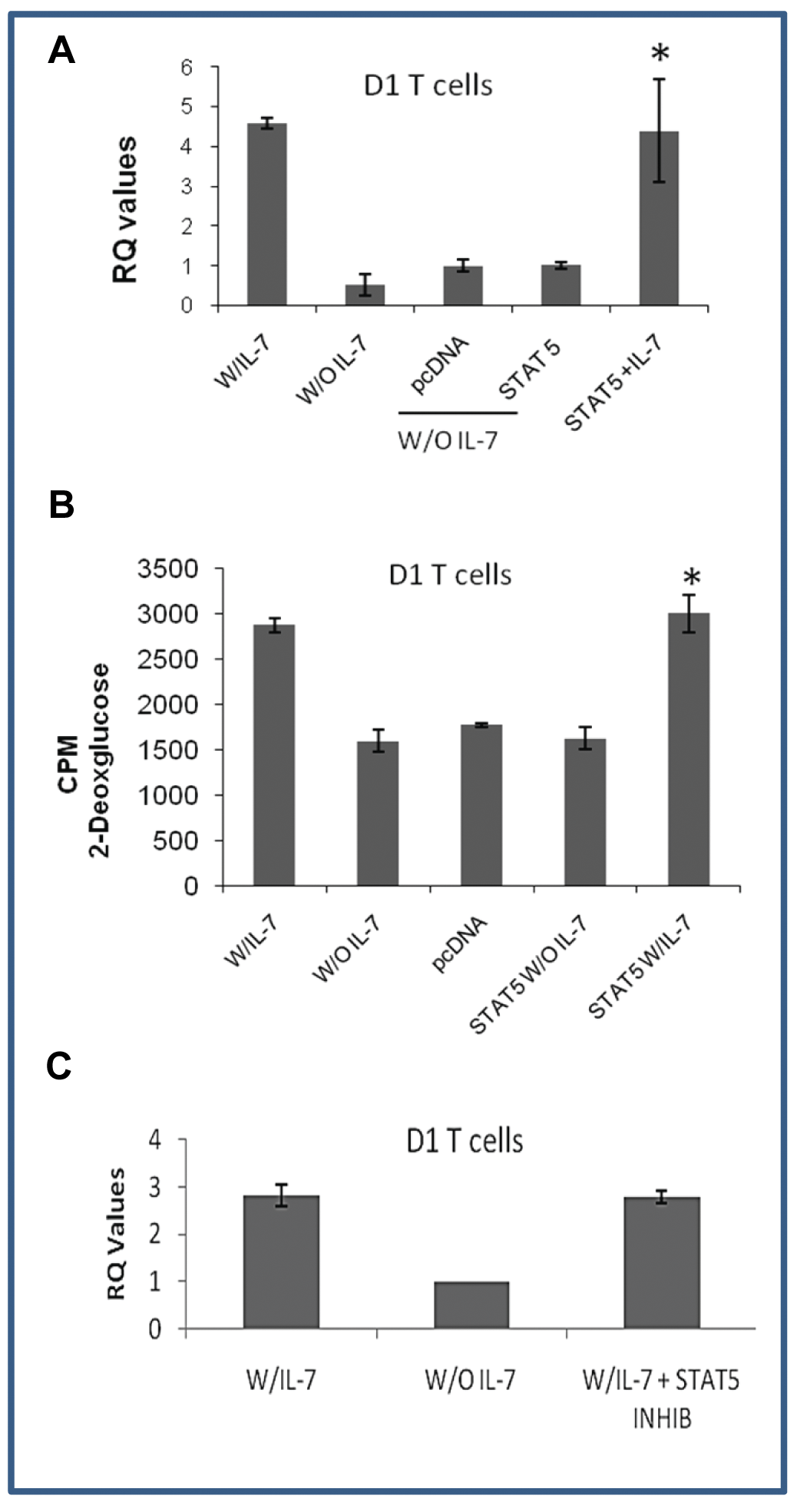

Supplement: Figure S1 — STAT5 does not promote HXKII gene expression or glucose uptake in response to IL-7. (A, B) D1 cells were nucleofected with pcDNA (empty vector) or constitutively active STAT5a-CA (A) and evaluated for HXKII gene expression using quantitative PCR or (B) glucose uptake as described in Methods. Results are representative of two independent experiments performed in triplicates (values in graphs are mean ± SD). (C) Quantitative PCR evaluation of HXKII gene expression in D1 cells after culture with or without IL-7 for 18 hours, or with IL-7 in the presence of a STAT5 inhibitor (50 µM) for 18 hours. RQ (Fold change in gene expression normalized to β-actin) = 2−ΔΔCt. (*) indicates P value of <0.05. (TIF) [file pone.0032262.s001.tif]

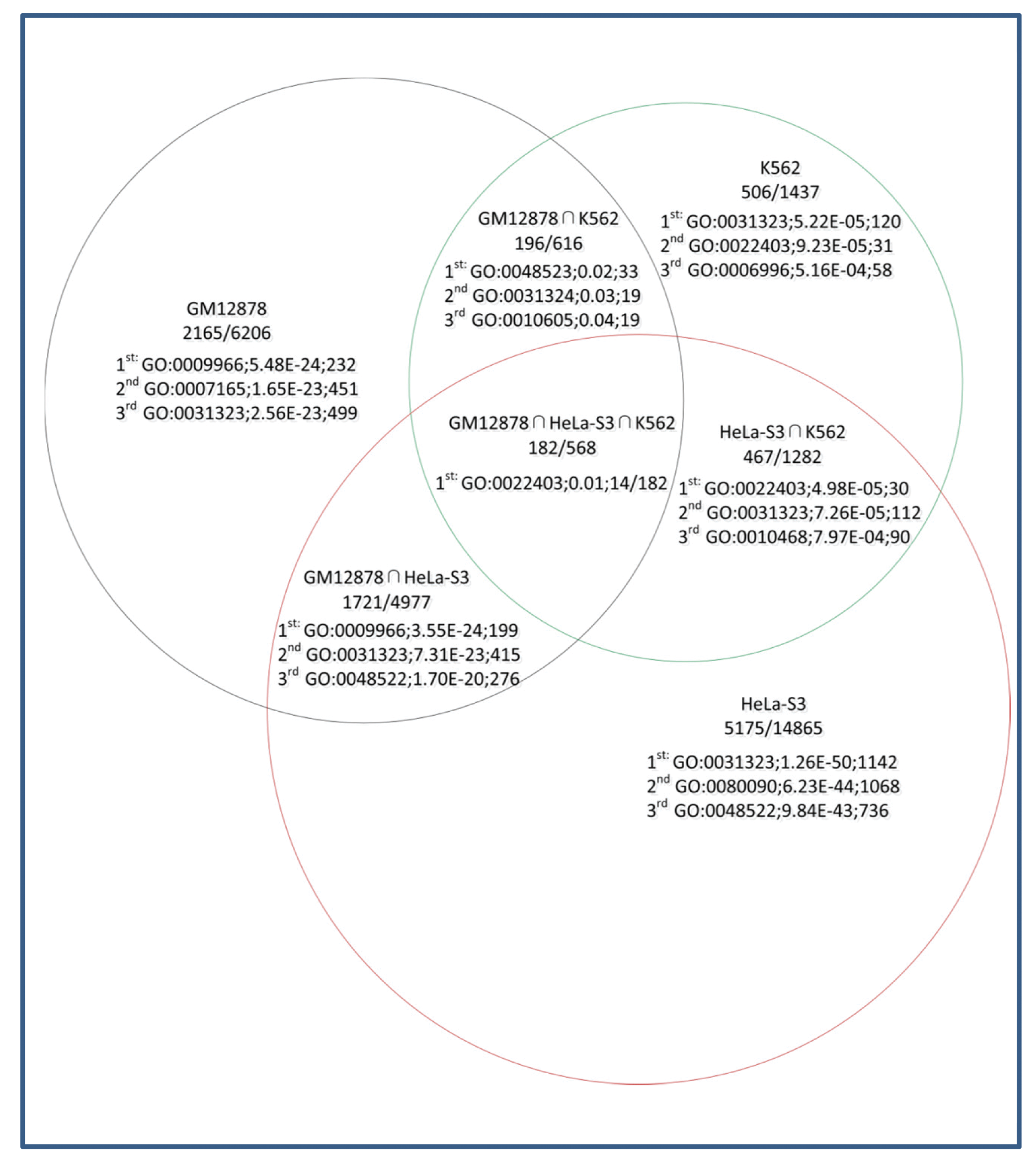

Supplement: Figure S2 — Identified JunD target genes in the three cell lines. JunD-regulated gene targets were identified by ChIP-seq as described in Methods. The top three most significant gene ontology terms that are at least at level 5 for each group of target genes are provided when they are available. For each term, a gene ontology term ID is provided, followed by the p-value of significance, and the number of target genes annotated with the term. The two numbers under each cell line name are the number of annotated target genes and the number of target genes for the specific cell line. (TIF) [file pone.0032262.s002.tif]
